# Association between DNA Methylation in the Core Promoter Region of the CUT-like Homeobox 1 (*CUX1*) Gene and Lambskin Pattern in Hu Sheep

**DOI:** 10.3390/genes14101873

**Published:** 2023-09-26

**Authors:** Xiaoyang Lv, Yue Li, Weihao Chen, Shanhe Wang, Xiukai Cao, Zehu Yuan, Tesfaye Getachew, Joram Mwacharo, Aynalem Haile, Yutao Li, Wei Sun

**Affiliations:** 1 Joint International Research Laboratory of Agriculture and Agri-Product Safety of Ministry of Education of China, Yangzhou University, Yangzhou 225009, China; 2 International Joint Research Laboratory in Universities of Jiangsu Province of China for Domestic Animal Germplasm Resources and Genetic Improvement, Yangzhou University, Yangzhou 225009, China; 3 College of Animal Science and Technology, Yangzhou University, Yangzhou 225009, China; 4 Animal Husbandry and Veterinary Station, Zhuba Street, Hongze District, Huai’an 223100, China; 5 International Centre for Agricultural Research in the Dry Areas, Addis Ababa 999047, Ethiopia; 6 CSIRO Agriculture and Food, 306 Carmody Rd., Saint Lucia, QLD 4067, Australia; yutao.li@csiro.au; 7 “Innovative China” “Belt and Road” International Agricultural Technology Innovation Institute for Evaluation, Protection, and Improvement on Sheep Genetic Resource, Yangzhou 225009, China

**Keywords:** sheep, *CUX1*, expression, DNA methylation, lambskin pattern

## Abstract

CUT-like homeobox 1 (*CUX1*) has been proven to be a key regulator in sheep hair follicle development. In our previous study, *CUX1* was identified as a differential expressed gene between Hu sheep lambskin with small wave patterns (SM) and straight wool patterns (ST); however, the exact molecular mechanism of *CUX1* expression has been obscure. As DNA methylation can regulate the gene expression, the potential association between *CUX1* core promotor region methylation and lambskin pattern in Hu sheep was explored in the present study. The results show that the core promoter region of *CUX1* was present at (−1601–(−1) bp) upstream of the transcription start site. A repressive region (−1151–(−751) bp) was also detected, which had a strong inhibitory effect on *CUX1* promoter activity. Bisulfite amplicon sequencing revealed that no significant difference was detected between the methylation levels of *CUX1* core promoter region in SM tissues and ST tissues. Although the data demonstrated the differential expression of *CUX1* between SM and ST probably has no association with DNA methylation, the identification of the core region and a potential repressive region of *CUX1* promoter can enrich the role of *CUX1* in Hu sheep hair follicle development.

## 1. Introduction

Hu sheep is a native breed of sheep characterized by precocity and high productivity in China. Hu sheep lambskins, known for their unique water-wave pattern, are popular in the clothing industry and the home sector worldwide [1]. The type of wave pattern is determined by many factors, such as curvature, density, and fineness of the wool, among which the pattern width is the major determination factor. According to the pattern width, Hu sheep lambskin can be classified into four grades: small waves (0.5–1.25 cm), medium waves (1.25–2 cm), large waves (≥2 cm), and straight hair (no pattern) [2]. Of these, the small wave pattern is the finest and is known as “Soft gemstones in China”. Mechanistically, the formation of the lambskin pattern is closely related to the hair follicle functionalities; after hair morphogenesis, the hair follicle undergoes cyclic transformation including growth (anagen), apoptosis-driven regression (catagen), and relative quiescence (telogen). The hair follicle’s functionalities are largely controlled by dermal papilla cells (DPCs), a kind of dermal origin cell composed of specialized fibroblasts of mesenchymal origin located in the center of the hair bulb, which function as a reservoir of multi-potent stem cells and specify hair size, shape and cycling [3]. Molecular studies to date have revealed several key transcriptional signaling mediating cellular progresses of DPCs, such as TGFβ [4], Wnt [5], Shh [6], Notch [7], BMP [8], etc. BMP and TGFβ are examples of prominently “suppression” pathways in hair growth. The downregulation of BMP- and TGF-related genes have been proven to be associated with the promotion of hair growth. The activation of Wnt is involved in the re-epithelialization of hair follicles and upgrades hair regrowth. Notch controls cell differentiation of hair follicle stem cells, ensuring the growth of the inner root sheath and hair shaft, while the activation of Shh can induce the proliferation of hair follicle stem cells and lead hair morphogenesis. In addition, subsets of candidate gene have also been identified, such as Sox10 in the hair follicle stem cell cycle [9], WNT10A in hair morphogenesis [10] and BMP7 in DPC proliferation [11]. Although subsets of transcriptional signaling and candidate genes have been revealed, the specific genetic mechanisms controlling the DPCs are still unknown, especially in sheep. CUT-like homeobox 1 (*CUX1*), which is also known as *CULT1*, belongs to the homeodomain transcription factor family [12]. As a transcription factor, numerous studies on the transcriptional repressor role of *CUX1* have been reported, the promoter-specific CCAAT-displacement activity of *CUX1* can bind to the region surrounding the CCAAT box and repress the expression of the target protein even when the protein is bound at a distance. To date, *CUX1* has been proven to participate in many biological processes [13,14,15], including tissue development, diverse cellular processes, DNA damage repair, etc. Regarding hair follicle functionalities, previous studies in mice showed that the inactivation of *CUX1* can lead to altered hair follicle morphology, and the loss of its exon (*CUX1*ΔCR1, also known as *CUX1* ^tm1Ejn^) can disturb the proper growth of the hair fiber and cause multiple hair abnormalities. The mutant *CUX1* ^tm1Ejn^ can also lead to coat hair in mice, while the abnormal hair phenotype diminishes with age [16,17]. Regarding the key pathways regulating hair follicle functionalities, several lines of evidence have shown that *CUX1* can serve as a positive regulator of the Wnt pathway in cancers [18]. A conserved *CUX1*-binding site exists in wnt5a, a key gene regulating TGFβ and Notch signaling [19], and although no direct evidence has proven a connection between these and *CUX1* in hair follicle growth, these aforementioned results enlighten us that certain regulation may also exist in the sheep hair follicle functionalities. The effect of *CUX1* on sheep lambskin has also been reported in our previous studies; Zhou et al. [20] revealed that the overexpression of *CUX1* can drive the proliferation of DPCs and regulate the activation of subsets of genes including *WNT10*, *MMP7*, *C-JUN*, *PPARD*, *FOSL1,* etc. (key genes in Wnt/β-catenin pathway functions essentially in hair follicle induction) [21]. In addition, we also found that miR-143 can function as a sponge to *CUX1* to downregulate its mRNA and protein expression, and consequently affect the proliferation of DPCs [22], which increases our understanding of *CUX1* expression regulation and hair follicle growth and development. Collectively, this evidence led to our hypothesis that the sheep hair follicle functionalities are mediated by *CUX1* expression; however, the specific molecular mechanism is still required to be deciphered.

DNA methylations are one of the most common epigenetic modifications in promoter regions of eukaryotic genomes. They are involved in gene silencing and genetic stability in mammals [23,24,25]. Regarding hair follicle functionalities, the methylation statuses of several key transcripts have also been proven to be biomarkers in hair development. For example, Kim et al. [26] reported that the methylation level of the hair *CSX* and *SOX* genes increased with age before 2 years old in Angora rabbits [27], and the CpG5 (−175 bp) site of Wnt10b serves as a critical methylated site in the secondary hair follicle cycle. DNA methylation of non-coding transcripts can also mediate hair development. Zhao et al. found that methylation levels of the lncRNA2919 promoter were involved in rabbit hair follicle regeneration [28]. The lncRNA-related DNA methylation level was found to be lower in the differentiation stage compared to the induction stage in Cashmere goat hair follicles [29]. A previous study conducted by Laurent et al. [30] found that DNA methylation at the *CUX1* gene is closely related to early-life growth trajectories and male infertility. Considering that the formation of lambskin is determined during the early-life growth period of lambs, and that *CUX1* was found differentially expressed in the hair follicles of lambskin with small waves and straight hair patterns [31], a hypothesis was thus raised that the DNA methylation on *CUX1* may be the reason for the regulation of *CUX1* expression in sheep lambskin. Our goals were thus to investigate the association between DNA methylation in the *CUX1* gene and Hu sheep lambskin pattern. In the present study, we first evaluated the expression of the *CUX1* gene and Hu sheep lambskin tissue and determined the core promoter region using bioinformatics analyses and dual luciferase assay. Then, the methylation level of the *CUX1* core promoter region was detected via bisulfite amplicon sequencing. Our study aims to gain insights into the *CUX1*-related molecular mechanism underlying hair follicle functionalities, and provide basic knowledge for lambskin production in the sheep industry.

## 2. Material and Methods

### 2.1. Sample Collection

The sample information was detailed described in our previously published reports [11]. Briefly, all experimental lambs were supplied by Suzhou Stud Farm (Suzhou, China). Approximately 1 cm^2^ of skin from the dorsal side of full-sib 3-day-old Hu sheep lambs with different lambskin patterns was collected and divided into two groups: small waves (SM, *n* = 3), and straight wool (ST, *n* = 3). The collected samples were snap-frozen in liquid nitrogen and then stored at −80 °C until use.

### 2.2. Quantitative Real-Time PCR

Total RNA was extracted from the collected tissues using Trizol (TIANGEN, Beijing, China) per the manufacturer’s instructions. The first strand of cDNA was prepared using a FastKing RT Kit according to the manufacturer’s instructions (TIANGEN, Beijing, China), and cDNA was stored at −20 °C until use.

Quantitative real-time PCR (RT-qPCR) was performed in 20 μL of reaction mixture that contained 10 µL FastReal qPCR premix (TIANGEN, Beijing, China), 0.4 µL of each forward and reverse primer, 6.4 µL RNase-Free ddH_2_O, and 2 µL cDNA. PCR amplification was performed in triplicate using the following conditions: initial denaturation at 95 °C for 5 min, followed by 40 cycles of 95 °C for 10 s, and 60 °C for 30 s. The dissociation curve was analyzed after amplification. A melting temperature (Tm) peak at 85 °C ± 0.8 on the dissociation curve was used to determine the specificity of PCR amplification.

*GAPDH* was selected as the reference gene, the primers were designed using Primer Premier 5 software (Supplementary Table S1). The 2^−ΔΔCt^ method [32] was used to calculate the expression level of *CUX1*. The results were shown as relative expression level (log_2_ Fold Change mean ± standard error) using GraphPad Prism 6 software.

### 2.3. Cell Culture

Isolation, culture and identification of Hu lamb dermal papilla cells (DPCs) were carried out according to our laboratory methods [33]. DPCs and HEK-293T cell line were cultured in DMEM/F12 (Gibco, Grand Island, NY, USA) supplemented 10% fetal bovine serum (Sigma, St. Louis, MO, USA), penicillin (100 U/mL) and streptomycin (100 mg/mL) with 5% CO_2_ at 37 °C.

### 2.4. Determination of CUX1 Core Promoter Region

The 2000-bp upstream sequence of the transcription start site of the sheep (*Ovis aries*) *CUX1* gene was obtained from the NCBI database (Accession number: XM_004020987.5). The prediction of the *CUX1* core promoter region was conducted using Neural Network Promoter Prediction software v2.0 (https://www.bdgp.org/seq_tools/promoter.html, accessed on 1 June 2023). Based on the prediction results, the 2000-bp upstream sequence was truncated at the 3′ end for the amplification of six promoter fragments including *CUX1*-U1 (1985 bp), *CUX1*-U2 (1601 bp), *CUX1*-U3 (1151 bp), *CUX1*-U4 (751 bp), *CUX1*-U5 (499 bp) and *CUX1*-U6 (149 bp). The detailed information on the designed primers is shown in Supplementary Table S1.

Then, the genomic DNA was isolated from the collected skin tissues using the TIANamp genomic DNA Kit (TIANGEN, Beijing, China) as per the manufacturer’s instructions. The six promoter fragments were amplified from the isolated genomic DNA using PCR, and then gel-purified, sequenced and ligated into a pGL3-Basic vector using ClonExpress^®^ II One Step Cloning Kit (Vazyme, Nanjing, China).

Subsequently, pGL3-*CUX1*-U1~U6 were co-transfected with a pRL-TK vector into DPCs and HEK-293T using jetPRIME transfection reagent (Polyplus-transfection, Illkirch-Graffenstaden, France). The cells were collected at 48 h after transfections for the dual luciferase assay using the Dual Luciferase Reporter Assay Kit (Vazyme, Nanjing, China) as per the manufacturer’s instructions. The *CUX1* core promoter region was determined based on the luciferase activity of each sample normalized to the Renilla luciferase activity.

### 2.5. DNA Methylation Detection in CUX1 Core Promoter Region

The Genomic DNA of skin tissues was transformed using the EpiTect Fast DNA Bisulfite Kit (QIAGEN, Hilden, Germany), the transformed DNA was used as a template for PCR amplification via the PyroMark PCR Kit (QIAGEN, Hilden, Germany). The PCR primers (Supplementary Table S1) were designed based on the sequence of sulfite transformation using the MethPrimer software v2.0 (http://www.urogene.org/cgi-bin/methprimer2/MethPrimer.cgi, accessed on 1 July 2023). Then, the PCR products were purified and ligated into the pMD19-T vector.

Ten positive clones of SM and ST groups were randomly chosen for bisulfite amplicon sequencing (Tsingke Biotech Co., Ltd., Beijing, China). The methylation level was evaluated using the QUMA software v1.1.13 (http://quma.cdb.riken.jp/, accessed on 1 July 2023).

### 2.6. Statistical Analyses

All statistical analyses were conducted using the SPSS 18.0 software (Chicago, IL, USA). Student’s *t*-test was used to detect the difference between the SM and ST groups. *p* < 0.05 was considered as statistically significant difference.

## 3. Results

### 3.1. CUX1 Was Identified as a Potential Candidate Gene Regulating Lambskin Pattern of Hu Sheep

**Figure 1:**
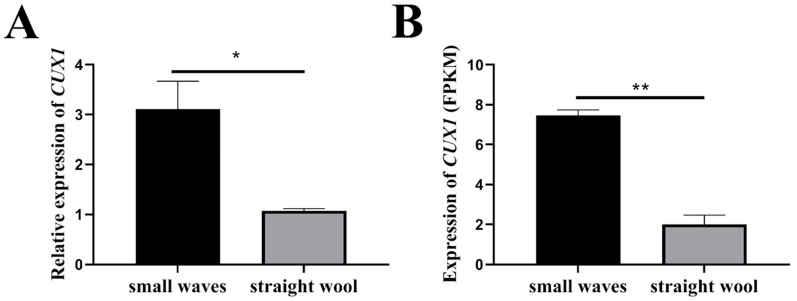
Expression of *CUX1* in lambskin tissues with SM pattern and ST pattern assessed with RT-qPCR (**A**) and RNA-seq (**B**). Note: * *p* < 0.05, ** *p* < 0.01.

In our previous study [31], we conducted an RNA-seq study to detect the mRNA expression in the hair follicles of Hu sheep lambskin with SM pattern and ST pattern, within the differentially expressed genes, *CUX1* was found to be highly expressed in the hair follicles of the SM group (Figure 1B) and was considered as a potential candidate gene regulating the lambskin pattern of Hu sheep.

In the present study, we further evaluated the expression of *CUX1* in lambskin tissues with SM and ST patterns (Figure 1A). The results demonstrate that the expression of *CUX1* was >6 fold higher in SM than ST, which indicates that *CUX1* expression was significantly higher in the lambskin tissue of SM than in ST (*p* < 0.05).

### 3.2. Determination of CUX1 Core Promoter Region

Considering that the promoter regions are the main regulatory elements of gene expression, our study mainly focused on the core promoter-induced *CUX1* expression mechanisms underlying lambskin pattern formation.

**Figure 2:**
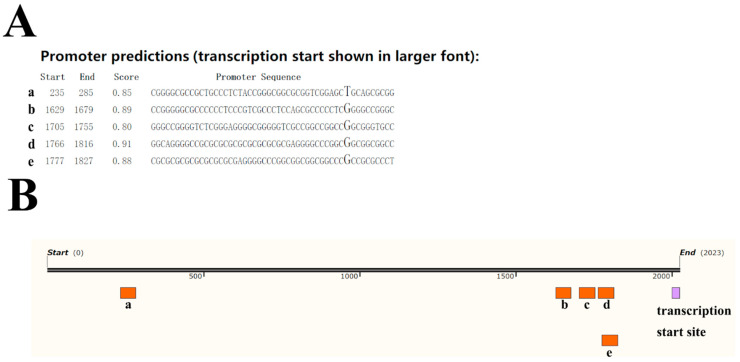
Prediction result of core potential promoter regions (**A**) and their locations (a~e) on the upstream region of the *CUX1* transcription start site (**B**).

First, a 2023-bp sequence upstream of the *CUX1* transcription start site was obtained and assessed using the Neural Network Promoter Prediction software. The results showed that five potential core promoter regions of the *CUX1* gene were predicted, which were present at −1789–(−1739) bp (a), −395–(−345) bp (b), −319–(−269) bp (c), −258–(−208) bp (d) and −247–(−197) bp (e) upstream of the transcription start site (Figure 2).

**Figure 3:**
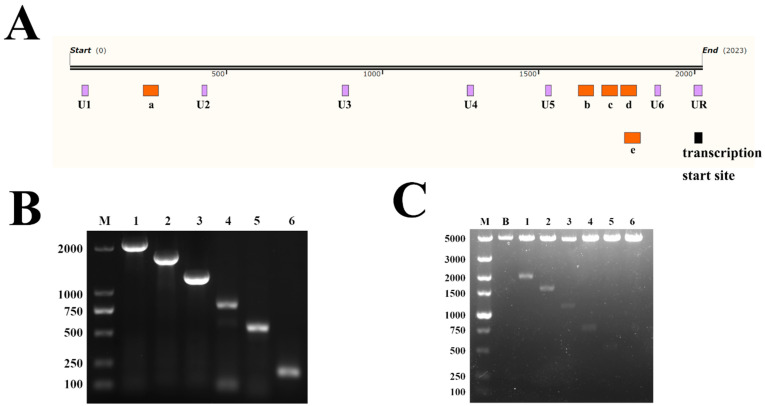
(**A**) Location of U1~U6 on the upstream region of the *CUX1* transcription start site. (**B**) The PCR amplification products of U1~U6, where M represents the DL2000 marker, 1~6 represent U1~U6. (**C**) pGL3-U1~U6 were confirmed via double digestion with SacI and HindIII, where M represents the DL5000 marker, B represents pGL3-Basic, and 1~6 represent pGL3-U1~U6.

Then, the 2023-bp upstream sequence was divided into six fragments according to the predicted potential core promoter regions (Figure 3A), containing −1985–(−1) bp (U1), −1601–(−1) bp (U2), −1151–(−1) bp (U3), −751–(−1) bp (U4), −499–(−1) bp (U5), −149–(–1) bp (U6). As shown in Figure 3B, the PCR amplification products of each fragment (Figure 3B) were evaluated via agarose gel electrophoresis and ligated into a pGL3-Basic vector. The results of the agarose gel electrophoresis showed that all six designed regions were successfully amplified and ligated into the vector (Figure 3C).

**Figure 4:**
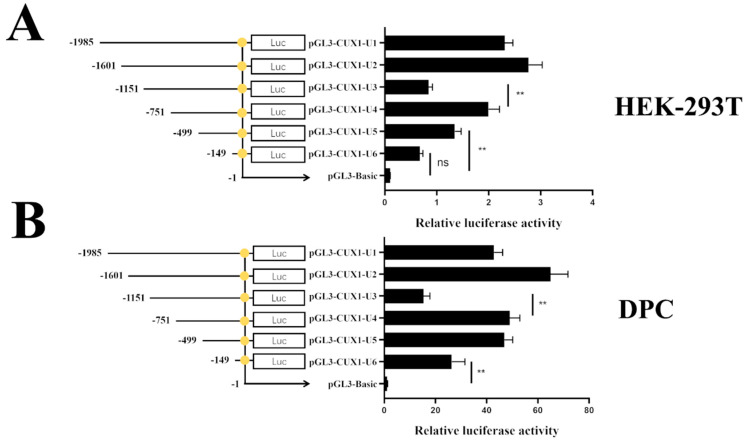
Relative luciferase activity of different vectors in HEK-293T (**A**) and DPC (**B**). Note: ** *p* < 0.01, ns: no significance.

Subsequently, pGL3-*CUX1*-U1~U6 and the pRL-TK vector were co-transfected into DPCs and HEK-293T for the dual luciferase assay. The results show that the luciferase activity of the U2 region was highest among all the regions both in DPCs (Figure 4A) and HEK-293T (Figure 4B), and luciferase activity was significantly decreased in the transformation of pGL3-*CUX1*-U1 and -U2, which indicates that the core promoter region of *CUX1* is located at −1601–(−1) bp.

It is worth noting that the luciferase activity of the pGL3-*CUX1*-U3 was extremely significantly lower (*p* < 0.01) than that of U4 and U6, and given the molecular mechanism of gene promotion, we assume that certain regulatory elements of *CUX1* transcription may exist at −1151–(−751) bp, such as the key methylated CpG sites.

### 3.3. DNA Methylation Detection in CUX1 Core Promoter Region

**Figure 5:**
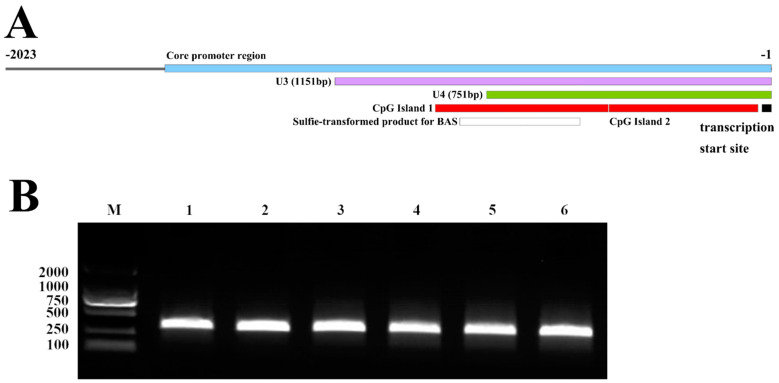
(**A**) Locations of predicted CpG islands on the upstream region of the *CUX1* transcription start site, where the red rectangle represents predicted CpG islands, the green rectangle represents U4, the purple rectangle represents U3, the blue rectangle represents the core promoter region (U5). (**B**) Sulfite-transformed products for the BAS, where M represents the DL2000 marker, 1~6 represent sulfite-transformed products.

To further explore the molecular regulatory roles of the CUX1 core promoter region, a methylation analysis was conducted. The prediction results of MethPrimer (Figure 5A) showed that two CpG islands exist in the CUX1 core promoter region, namely, −886–(−429) bp (Island 1) and −427–(−33) bp (Island 2). Considering the overlap between the U3 region and the predicted CpG islands, CpG Island 1 was selected and −821–(−505) bp of Island 1 region was sulfite-transformed (Figure 5B) for bisulfite amplicon sequencing (BAS).

**Figure 6:**
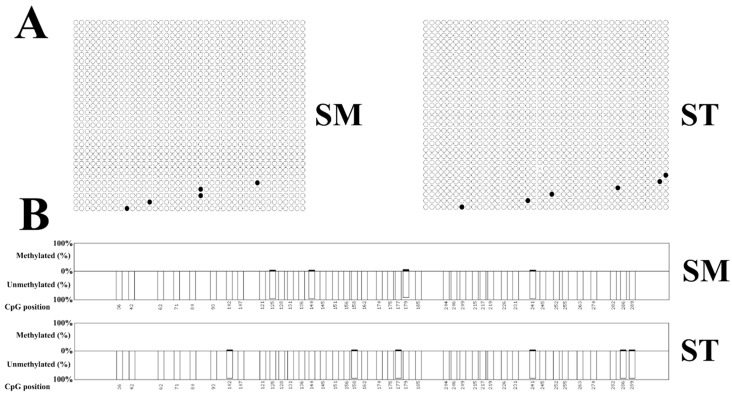
The results of BAS (**A**,**B**) locations of methylated CpG sites on the selected region, where black nodes represent methylated CpG sites, white nodes represent unmethylated sites.

The results of BAS (Supplementary Material S2) showed that only four and six methylated CpG sites were detected in the selected region of SM tissues and ST tissues under 30 repetitions, respectively (Figure 6). The average methylation levels of SM and ST were 0.9% and 1.0%, respectively. No significant difference was detected between the methylation levels of the SM group and the ST group, indicating that there was no statistical correlation between the DNA methylation and *CUX1* mRNA expression.

## 4. Discussion

The formation of lambskin pattern is a dynamic and complex process which happens in early life and involves delicate molecular regulatory mechanisms. Lambs carrying hairy lambskin (with waves) are of more economic value and have proven to be more adapted to harsh environmental conditions. Although the genetic basis of Hu sheep lambskin pattern formation is still largely unknown, studies on other sheep breeds have identified subsets of candidate genes in wool phenotype; a genome-wide study in Romane and Merinos sheep revealed that the insertion of a polymorphism (asEIF2S2) into the 3′ UTR of the *IRF2BP2* gene can lead to the regulation of *EIF2S2* and *IRF2BP2* mRNA expression, and is associated with the formation of hairy fleece and woolly fleece phenotypes [2]. Interestingly, the *EIF2S2* and *IRF2BP2* genes have both proven to be a regulator of transcription factors (*EIF2S2* encodes a transcription factor and *IRF2BP2* functions as a transcription factor coregulator). Another well-studied transcription factor that plays essential roles in hair follicle functionalities is *SOX10.* As a member of the Sox gene family, *SOX10* can mediate the expression of genes involved in stem cell behavior during embryonic development. During the hair follicle cycle, the expression of *SOX10* can control the hair follicle stem cell function and regulate hair follicle morphogenesis and postnatal follicular cycling [9]. In addition, other transcription factors such as *HOXC13*, *JUNB*, *LHX2*, *VDR* and *GATA3* have also been reported participating in hair follicle differentiation [29]. Collectively, the unravelling of the crucial function of transcription factors in wool pattern formation gives rise to our interest in transcription factor-regulated mechanisms in Hu sheep lambskin phenotype. *CUX1* is a key member of the homeodomain transcription factors family. According to its specific CCAAT-displacement activity, *CUX1* can function as a transcriptional repressor during the cell cycle and has proven to be associated with multiple cellular processes including cell differentiation, proliferation, cell cycle, etc. [34,35]. Our previous study established that an increased level of *CUX1* expression is associated with the proliferation of DPCs, which can lead to the promotion of hair follicle growth and development in sheep. Several studies have reported post-translational modifications of *CUX1* including phosphorylation, dephosphorylation, acetylation and proteolytic processing [36], which can lead to a decrease in DNA binding activity of *CUX1*; however, there is little knowledge concerning the other transcriptional modifications of *CUX1*, such as DNA methylation. Here, we analyzed the effects of promoter methylation on *CUX1* transcriptional activity and its potential role in the formation of lambskin pattern.

It is well acknowledged that DNA methylation is a fundamental epigenetic mark that correlates with gene expression. Regarding *CUX1*-related researches, the methylation modifications of *CUX1* have been proven to be associated with complex biological processes, including amygdala and hippocampus development [37], chronic kidney disease [38] and obesity [30]. In sheep, a increased methylated level of *CUX1* was observed in preterm ovine cerebrum and cerebellum compared to the normal ovine. *CUX1* was also identified as a highly methylated gene in brain tissue of fetal sheep [39], which highlighted the effect of *CUX1* methylation on early nervous system development; however, the role of *CUX1* methylation in sheep hair follicle growth and development remains obscure. Our previous study [31] detected mRNA expression in the hair follicles of Hu sheep lambskin with SM and ST patterns, and *CUX1* was found to be highly expressed in the hair follicles of the SM group. Consistent with our previous results, the present study also revealed this in the lambskin tissue of the SM group rather than the ST group, which further proves that *CUX1* is a potential candidate gene regulating the lambskin pattern of Hu sheep.

Promoter methylation is the most obvious epigenetic modification of DNA methylation [40,41,42]. The core promoter is a part of the promoter region which can assemble the RNA polymerase and initiate transcription [43]. To explore the effect of DNA methylation on *CUX1* expression, the core promoter region of the *CUX1* gene was first defined via a dual luciferase reporter assay. Estimating promoter activity is the most commonly used approach to determine the core promoter region. In the present study, we observed that the U2 region (−1601–(−1) bp) had the highest luciferase activity in both the 293T cell line group and the DPCs group. The previous study showed that core promoter motifs can significantly increase promoter activity [44,45]; hence, the U2 region was considered as a core promoter region of *CUX1* in sheep. Generally, the luciferase activity of truncated core promoter fragments decreases gradually with increasing truncation length; however, the luciferase activity of the U3 (−1151–(−1) bp) was observed to be significantly lower than that of U2 and U6 (−751–(−1) bp). Similar results [46] were observed by Robin et al., who identified an important repressive region on the *lck* promoter which, when deleted, causes an increase in promoter activity and *lck* expression. Xu et al. also found that deterrent transcription factors exist in cattle; deletion of the *PAX7* gene promoter region can lead to a 2.79-fold increase in promoter activity [47]. Collectively, this evidence suggest that this region (−1151–(−751) bp) should contain repressive elements of *CUX1* promoter activity; deletion of this region may also have a promoting effect on the *CUX1* promoter activity and expression. Of course, in-depth analyses are needed to prove our hypothesis.

Subsequently, DNA methylation analyses were conducted based on the identified core promoter region. The results of the MethPrimer showed that two CpG islands were predicted, which were present at −886–(−429) bp (Island 1) and −427–(−33) bp (Island 2). Interestingly, the majority of Island 1 and Island 2 were present at U4 and U3 regions, not in the core promoter region (U2). Previous research has shown that methylation of key CpG sites can influence the promoter activity of a specific gene [48,49,50,51]; hence, we raised the hypothesis that certain methylated CpG sites also occurred in the aforementioned potential repressive region. Considering the overlapping region between predicted CpG islands and the potential repressive region, CpG Island 1 was selected for the BAS. Unfortunately, only four and six methylated CpG sites were detected in the SM tissues and ST tissues, respectively. No differential methylation level was detected in the *CUX1* core promoter region, which indicates that there was no correlation between the methylation of CpG sites in Island 1 and *CUX1* mRNA expression. As previously mentioned, *CUX1* was proven to be a highly methylated gene in the lamb nervous system [39]; however, our present study indicates that, in sheep hair follicles, the methylation machinery is dispensable for promoter activity. Whether DNA methylation regulates *CUX1* expression remains to be established. It is possible that the *CUX1* expression is regulated by other factors, such as miR-143, as we previous reported [22].

It is cautionary to mention the limitations of our study regarding the bisulfite amplicon sequencing and *CUX1* core promoter region. Considering the overlapping region between the predicted CpG Islands and the repressive region, only CpG Island 1 was selected for the BSA and the DNA methylation analyses showed no difference between the two groups; The results could have been different if CpG Island 2 was selected for the BSA. Regarding the determination of the *CUX1* core promoter region, to date, no study focused on the *CUX1* core promoter has been reported; hence, the required knowledge is lacking to make comparisons between different species, and further study is still required to validate the reliability of the U2 region as a *CUX1* core promoter in other species.

## 5. Conclusions

In conclusion, the expression level of *CUX1* was significantly higher in SM tissues than in ST tissues. Five potential core promoter regions of the *CUX1* gene were predicted based on bioinformatics analysis, and the core promoter region of *CUX1* was determined at (−1601–(−1) bp) upstream of the transcription start site via dual luciferase assay. A repressive region (−1151–(−751) bp) with a strong inhibitory effect on *CUX1* promoter activity was also identified. Bisulfite amplicon sequencing showed that four and six methylated CpG sites were detected in the *CUX1* CpG Island 1 region of SM tissues and ST tissues, respectively. No significant difference was detected between the methylation levels of SM tissues and ST tissues; hence, the differential expression of *CUX1* between SM and ST probably has no association with the *CUX1* core promoter DNA methylation. Our studies further enrich the role of *CUX1* in hair follicle growth and development and can provide basic knowledge for the Hu sheep lambskin industry.

## Data Availability

Not applicable.

## Supplementary Materials

The following supporting information can be downloaded at: https://www.mdpi.com/article/10.3390/genes14101873/s1, Supplementary Table S1: Primers used in the present study. Supplementary Material S2: Raw results of bisulfite amplicon sequencing. Supplementary Table S3: Original blot results in the present study.
